# Uncovering Predictors of Lipid Goal Attainment in Type 2 Diabetes Outpatients Using Logic Learning Machine: Insights from the AMD Annals and AMD Artificial Intelligence Study Group

**DOI:** 10.3390/jcm12124095

**Published:** 2023-06-16

**Authors:** Davide Masi, Rita Zilich, Riccardo Candido, Annalisa Giancaterini, Giacomo Guaita, Marco Muselli, Paola Ponzani, Pierluigi Santin, Damiano Verda, Nicoletta Musacchio

**Affiliations:** 1 Department of Experimental Medicine, Section of Medical Pathophysiology, Food Science and Endocrinology, Sapienza University of Rome, 00161 Rome, Italy; davide.masi@uniroma1.it; 2 Mix-x SRL, 10015 Ivrea, Italy; 3 Associazione Medici Diabetologi, Giuliano Isontina University Health Service, 34149 Trieste, Italy; riccardocandido67@gmail.com; 4 UOSD Diabetology, Department of Exchange and Nutrition Diseases, Brianza Health Service, Pio XI Hospital, 20833 Desio, Italy; annalisa.giancaterini@gmail.com; 5 Diabetes and Endocrinology Unit, ASL SULCIS, 9016 Iglesias, Italy; giacomoguaita@gmail.com; 6 Rulex Innovation Labs, Rulex Inc., 16122 Genoa, Italy; marco.muselli@ieiit.cnr.it (M.M.); damiano.verda@gmail.com (D.V.); 7 Diabetes and Metabolic Disease Unit, ASL 4 Liguria, 16043 Chiavari, Italy; paola.ponzani@gmail.com; 8 Deimos, 33100 Udine, Italy; p.santin@e-deimos.it; 9 Associazione Medici Diabetologi, 20156 Milano, Italy; nicoletta.musacchio@gmail.com

**Keywords:** dyslipidemia, machine learning, artificial intelligence, type 2 diabetes, low-density lipoprotein cholesterol

## Abstract

Identifying and treating lipid abnormalities is crucial for preventing cardiovascular disease in diabetic patients, yet only two-thirds of patients reach recommended cholesterol levels. Elucidating the factors associated with lipid goal attainment represents an unmet clinical need. To address this knowledge gap, we conducted a real-world analysis of the lipid profiles of 11.252 patients from the Annals of the Italian Association of Medical Diabetologists (AMD) database from 2005 to 2019. We used a Logic Learning Machine (LLM) to extract and classify the most relevant variables predicting the achievement of a low-density lipoprotein cholesterol (LDL-C) value lower than 100 mg/dL (2.60 mmol/L) within two years of the start of lipid-lowering therapy. Our analysis showed that 61.4% of the patients achieved the treatment goal. The LLM model demonstrated good predictive performance, with a precision of 0.78, accuracy of 0.69, recall of 0.70, F1 Score of 0.74, and ROC-AUC of 0.79. The most significant predictors of achieving the treatment goal were LDL-C values at the start of lipid-lowering therapy and their reduction after six months. Other predictors of a greater likelihood of reaching the target included high-density lipoprotein cholesterol, albuminuria, and body mass index at baseline, as well as younger age, male sex, more follow-up visits, no therapy discontinuation, higher Q-score, lower blood glucose and HbA1c levels, and the use of anti-hypertensive medication. At baseline, for each LDL-C range analysed, the LLM model also provided the minimum reduction that needs to be achieved by the next six-month visit to increase the likelihood of reaching the therapeutic goal within two years. These findings could serve as a useful tool to inform therapeutic decisions and to encourage further in-depth analysis and testing.

## 1. Introduction

Type 2 diabetes mellitus (T2DM) is one of the most common metabolic disorders worldwide and is associated with a high mortality rate due to increased cardiovascular (CV) risk [1]. However, taken alone, poor glycaemic control cannot entirely explain the increased risk of developing atherosclerotic cardiovascular disease (ASCVD) in diabetic patients [2]. Among additional risk factors, dyslipidemia has been reported to play a crucial role [3]. Approximately 70% of patients with T2DM have dyslipidemia [4], a spectrum of lipid alterations including increased triglycerides and low-density lipoprotein cholesterol (LDL-C) and reduced levels of high-density cholesterol (HDL-C) [5].

Lowering LDL-C through either lifestyle changes or pharmacologic therapy has been shown to reduce the risk of ASCVD in individuals with T2DM and to induce beneficial effects that are at least equal to those obtained in non-diabetic subjects, resulting in a significant reduction in total mortality and CV events [6,7]. In addition, the American Heart Association guidelines emphasize the importance of personalizing hypolipidemic therapy considering the enormous heterogeneity in CV risk, comorbidity burden, and life expectancy of patients with T2DM [8]. Over the years, the recommended LDL-C goals for diabetic patients have undergone several changes. Until 2017, the LDL-C level for these patients was recommended to be below 100 mg/dL (2.60 mmol/L), with the optional goal of achieving LDL-C levels below 70 mg/dL (1.8 mmol/L) in those with ASCVD [9]. However, in recent times, the LDL-C targets have become even more specific and stringent [10]. The most recent guidelines of the European Society of Cardiology (ESC) recommend a target LDL-C of 55 mg/dL (1.4 mmol/L) or lower and a minimum reduction in LDL-C >50% from baseline levels in all T2DM patients with established ASCVD [11].

Despite advances in treatment options, the response to lipid-lowering interventions is not uniform and only 66% of diabetic patients achieve LDL-C levels < 100 mg/dL (2.60 mmol/L), consequently the residual CV risk remains high [12].

Recent studies have investigated why some people with T2DM fail to achieve the expected LDL-C lowering despite maximal statin therapy. Genome-wide association studies have identified genetic variations, such as single nucleotide polymorphisms, that may contribute to the variability in hypolipidemic treatment response. [13] Other possible explanations include poor compliance with therapy and lifestyle recommendations. In this regard, the PROSPER study, conducted by Trompet and colleagues, suggests that among patients undergoing pravastatin treatment, non-responders are more likely to be non-adherent [14]. Additionally, recent research has proposed that gut microbiota may also influence the response to statin therapy in patients with dyslipidemia [15].

Despite previous research efforts, identifying the factors that contribute to lipid goal achievement remains an unmet clinical need.

In recent times, by combining network biology and machine learning approaches, valuable insights have been gained regarding potential drugs that could be novel viable options for treating dyslipidemia [16]. Additionally, this innovative approach has also been adopted for the identification of new predictors of cardiovascular risk in patients with T2DM [17]. Still, to our knowledge, no studies have used artificial intelligence (AI) techniques to analyse real-world data to determine which factors are most likely to predict therapeutic success.

In this study, we leveraged a machine-learning approach to identify variables that may potentially predict therapeutic success (or failure) when managing dyslipidemia in patients with T2DM. Specifically, the following two aims were proposed:

(1) To describe the characteristics of a large cohort of T2DM patients at the time of initiation of lipid-lowering therapy through a rigorous collection of anthropometric, clinical, and metabolic data while also providing the stratification of patients over the years, according to LDL-C range and CV risk grade;

(2) To evaluate the performance of the Logic Learning Machine (LLM) method in predicting the response to lipid-lowering therapy in T2DM patients, using clinical data from the large dataset (Annals) of the Italian Association of Medical Diabetologists (AMD).

## 2. Materials and Methods

### 2.1. Study Design and Eligibility Criteria

This is a retrospective, cross-sectional, longitudinal study based on data extracted from the AMD Annals database, which includes the electronic medical records of all patients treated in 271 Italian diabetes clinics between 2005 and 2019, as already discussed in our previous articles [18,19]. This study relied on pre-existing data and did not require the direct involvement of patients. The data of the patients were coded and stored anonymously.

#### 2.1.1. Inclusion Criteria

In the machine-learning analysis, we included all diabetic patients in the database who met the following inclusion criteria: (1) the time at which the cholesterol-lowering drug was started is known (defined as ‘T0’); (2) a measurement of LDL-C at T0 is present in the database; (3) follow-up visits were performed six months and two years after the first prescription of cholesterol-lowering therapy (defined as ‘T6M’ and ‘T2Y’, respectively); (4) at T6M, a measurement of LDL-C is present; and (5) at T2Y the prescription of a cholesterol-lowering drug is still present, and measurement of LDL-C has been performed.

#### 2.1.2. Exclusion Criteria

The study excluded individuals who met any of the following criteria: (1) type 1 diabetes; (2) gestational diabetes; and (3) patients who initiated dyslipidemia treatment after mid-2017. The latter exclusion criterion was applied to ensure a minimum clinical observation period of two years from the start of therapy, given that the database contained information only up to mid-2019. As a result, the final database consisted of data on 1,186,247 patients with type 2 diabetes.

**Figure 1:**
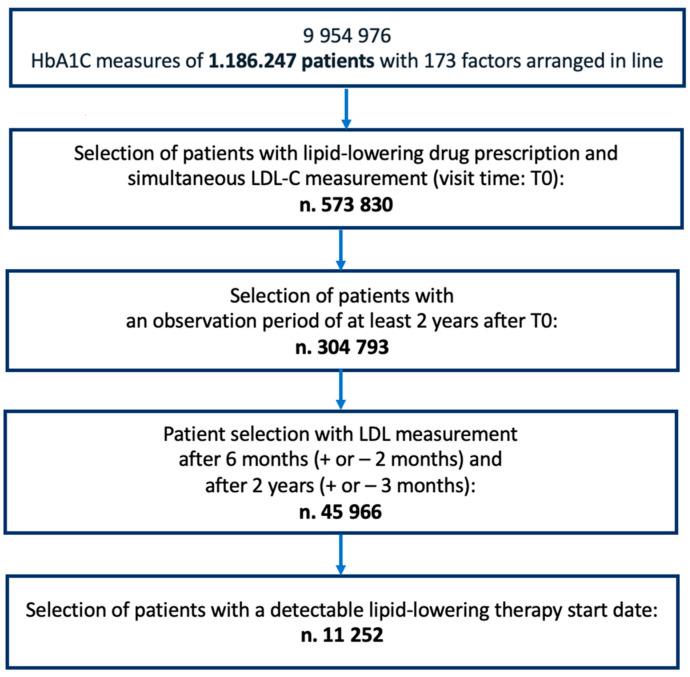
Flowchart regarding the selection of patients with type 2 diabetes who visited one of 271 diabetes centres between 2005 and 2019.

Figure 1 provides the flowchart of the selection of patients from the AMD database.

### 2.2. Database Description

The database included a record of 92 different variables (See Table S1 in Supplementary Materials), such as biochemical glucose and lipid metabolism parameters, as well as information regarding the type of statin and anti-diabetic treatment prescribed, but also clinical, anthropometric, organizational, and therapeutic parameters, as outlined in our previous works [20,21]. Another parameter included in the database is the Q-Score, an indirect index of the overall quality of healthcare at each centre, calculated using the achievement rate of specific targets (HbA1c, blood pressure, LDL-C, and microalbuminuria), as well as the appropriate prescription rate. The Q-Score has already been shown to predict the incidence of new major CV events in previous studies [22,23].

### 2.3. Lipid Target

According to recent ESC guidelines, patients were then stratified according to their CV risk [24]. Since the database included patients treated over a decade, using the most recent guidelines for LDL-C targets was not feasible due to significant changes in recommendations over time. Consequently, we applied the National Cholesterol Education Panel (NCEP) Adult Treatment Panel III (ATP III) guidelines [25] which recommended a minimum LDL-C target of 100 mg/dL (2.60 mmol/L) for diabetic patients with (high or) very high CV risk (i.e., patients with more than two risk factors and 10-year risk of 10–20%, or stage 3–4 chronic kidney disease (CKD) without other risk factors) [26].

### 2.4. LLM Characteristics and ML Modelling

In this study, we used Rulex^®^ (Innovation Lab, Rulex Analytics, 16122 Genova, Italy), a type of transparent LLM, which represents a cutting-edge supervised analysis technique built on the effective implementation of the switching neural network model. The specific characteristics of this LLM are summarized below.

Rulex^®^ utilizes the concept of ‘Shadow Clustering’, which is an aggregative policy wherein, during each iteration, a set of patterns that belong to the same output class are clustered together to form a comprehensible rule. Without any a priori knowledge, the LLM can create models selecting the most relevant variables that better explain an initial premise. The LLM subsequently makes explicit whether threshold values of the identified variables exist.

In detail, Rulex^®^ may identify a specific cut-off point for some variables, below/above which the probability of belonging to a specific class, or outcome, is inverted. In the case of continuous variables, the threshold value is a numerical value, while for qualitative variables, it represents the modality. However, there is not always a univocal probability reversal point, as some variables can contribute to the classification because they are present in the rules, but do not have a specific reversal point. When there is no univocal determination of the ‘threshold’ by the LLM, it is not made explicit in the modelling results. This LLM has recently been adopted in other research studies [20,27,28].

Thus, when employed in a prediction, Rulex^®^ makes explicit why for a new patient the response to the starting premise is yes or no. For our specific investigation, the two initial assumptions were as follows: the patient succeeds/fails in achieving the C-LDL goal (<100 mg/dL 2.60 mmol/L) by T2Y.

Furthermore, explainable machine learning leverages the augmented analytics (AA) paradigm, offering a unique advantage over other AI techniques. AA operates on the principle that additional information can be extracted from the data, which can then be used to improve the model’s predictive or explanatory power. To achieve this, the modelling cycles are repeated to verify the accuracy and efficacy of the dataset in explaining the phenomenon being analysed [29,30,31]. By integrating AA, Rulex^®^ provides a deeper understanding and interpretation of data, leading to novel insights that are both reliable and actionable.

In our LLM analysis, the AA principles were followed to select the most relevant factors related to target achievement. Initially, the LLM was only fed with patient characteristics at T0, which highlighted the strong dominance of the LDL-C value, but with only sufficient model performance. To improve the accuracy, another modelling was conducted, which added LDL-C trends after T0, resulting in a stronger model with high relevance of the added variable. To verify the result, the LDL-C trend before and after therapy was plotted for all patients divided into groups based on the initial LDL-C range (LDL-C < 75 mg/dL, 75–100 mg/dL, 100–125 mg/dL, 125–150 mg/dL, 150–175 mg/dL, 175–200 mg/dL, and >200 mg/dL) and the type of statin prescribed. The curves showed a systematic decrease in LDL-C in the first 6 months from the start of therapy. Based on this evidence, the final model was generated, which included the variation in LDL-C 6 months after T0 for each patient. Thus, the eligibility criteria described previously refer to the final model.

## 3. Results

### 3.1. General Patient Characteristics

The initial database consisted of 1.186.247 diabetic patients. Of these, 11.252 patients (45.46% female) with T2DM and dyslipidaemia were evaluated in this retrospective study. Stratification according to CV risk showed that, at T0, 97.7% of them were at very high risk and 2.3% at high risk, while at T2Y, 99.4% were at very high risk and 0.6% at high risk.

Furthermore, at T0, 95.79% of patients were prescribed statins (with or without ezetimibe) and only 9.16% ezetimibe (with or without statins; please refer to Figure S1 in the Supplementary Materials for more information).

Given the CV risk, the primary research goal was to achieve LDL-C levels below 100 mg/dL (2.60 mmol/L) within two years of the start of lipid-lowering therapy. This goal was achieved in 61.4% of the patients evaluated in the time interval ranging from 2005 to 2019.

Table 1 describes patients’ anthropometric and clinical characteristics at baseline (T0), at 6 months, and at 2-year follow-up (T2Y), broken down by success or failure in achieving the LDL-C target. To assess normality, we used the Jarque–Bera test. The Mann–Whitney test showed statistically significant differences (all *p* < 0.05) in age and baseline LDL-C, HDL-C, total cholesterol, uric acid, and eGFR between the patients reaching and the ones not reaching the LDL-C target at two years of therapy. No differences were found in fasting blood glucose, HbA1c levels, triglyceride concentrations, and blood pressure.

**Figure 2:**
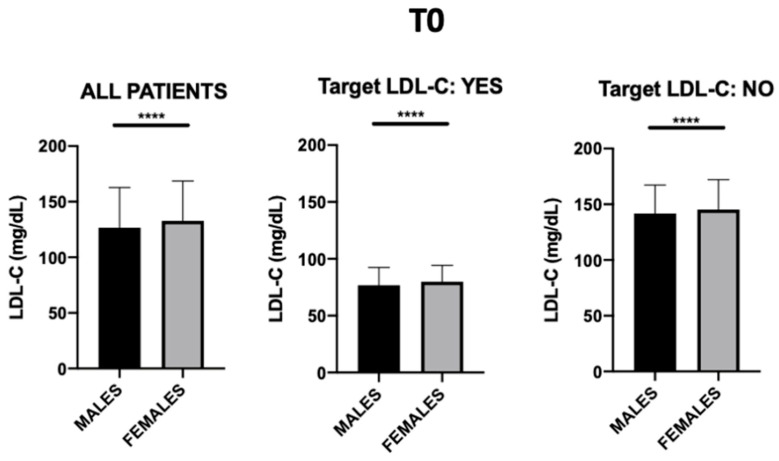
Gender differences in LDL-C at time T0, broken down by those who presented an LDL-C below 100 mg/dL and those who did not; **** *p*-value < 0.0001.

Regarding the gender distribution of our cohort, at baseline (Figure 2) the average LDL-C level for men was 126.5 mg/dL, whereas for women, it was 132.5 mg/dL. The results of Student’s *t*-test showed that the mean LDL-C level in women who were not at target at T0 was significantly higher than in men (145.2 vs. 141.8, *p* < 0.0001), indicating a gender difference in the risk of high LDL-C levels.

**Figure 3:**
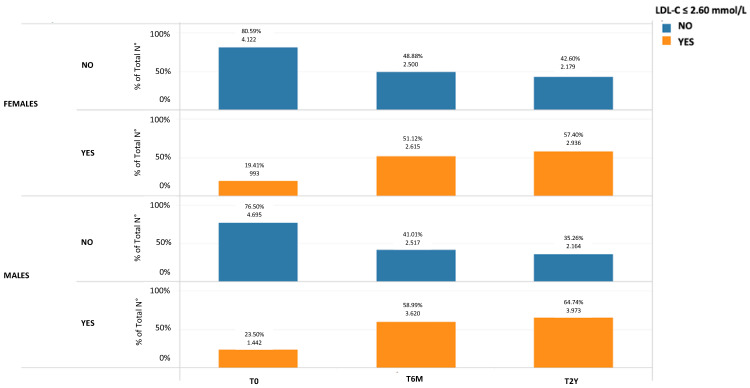
Gender differences in the number of patients presenting with an LDL-C lower than 100 mg/dL (2.60 mmol/L) at time T0, T6M, and T2Y.

Additionally, at T0, a higher proportion of women (80.59%) had LDL-C levels above 100 mg/dL (2.60 mmol/L) compared to men (76.50%). This gender difference becomes even more pronounced at time points T6M and T2Y, as shown in Figure 3. These findings suggest that women may have a poorer response to hypolipidemic therapy compared to men.

Subsequently, patients were grouped based on their LDL-C levels at the start of therapy into the following categories: LDL-C < 75 mg/dL, 75–100 mg/dL, 100–125 mg/dL, 125–150 mg/dL, 150–175 mg/dL, 175–200 mg/dL, and >200 mg/dL.

The percentages of patients for each LDL-C range related to T0, T6M, and T2Y are presented in Table 2.

**Figure 4:**
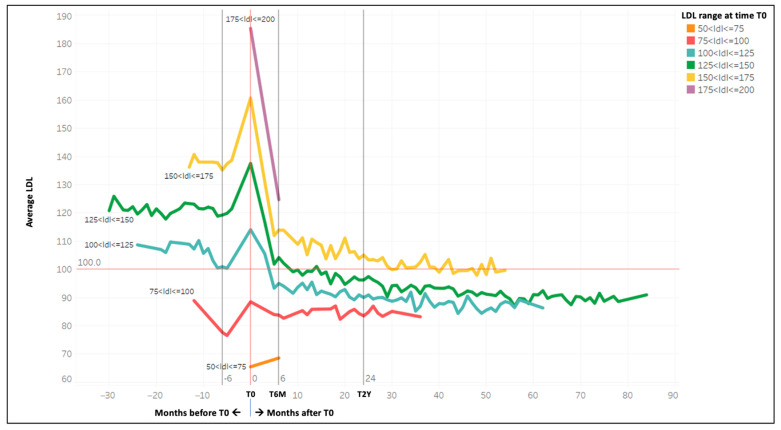
The trend of LDL-C averages of different patient groups according to the starting LDL-C range (with a minimum number of 200 patients for curve points).

**Figure 5:**
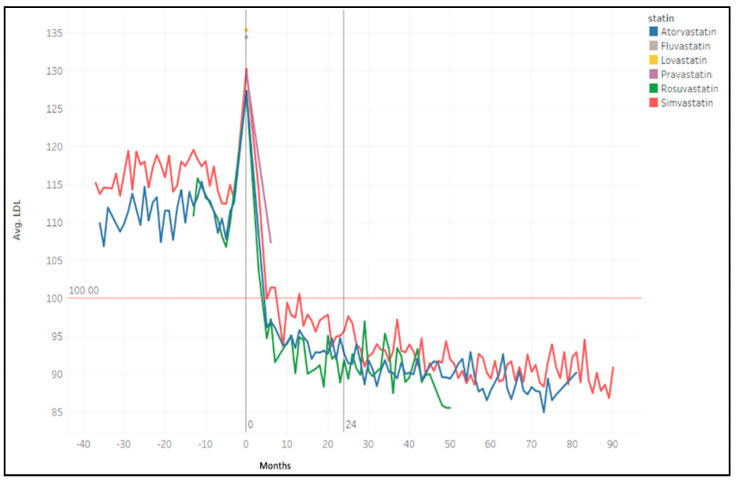
The trend of LDL-C averages of different patient groups according to different statin administration.

Figure 4 displays the average trends of LDL-C levels across all patient cohorts, both before and after T0, with a focus on the changes following the initiation of lipid-lowering therapy. Notably, the most significant reduction in LDL-C occurs within six months of starting treatment, regardless of the specific statin prescribed. Consequently, we included the LDL-C value at the six-month follow-up visit as one of the inclusion criteria for patient selection in the LLM analysis. Additionally, as shown in Figure 5, the trend in LDL-C levels remains consistent when stratifying by the type of statin administered to patients.

It is worth noting that when we evaluated trends in LDL-C levels, patients who achieved their target at T2Y continued to experience a decline in LDL-C levels even after T6M, whereas those who failed to reach their target exhibited a rise in values after T6M (please refer to Figure S2 in the Supplementary Materials for more information). These findings emphasize the significance of reviewing patients 6 months after initiating hypolipidemic therapy to consider modifying treatment plans for those who do not achieve their LDL-C target.

### 3.2. LLM Analysis

**Figure 6:**
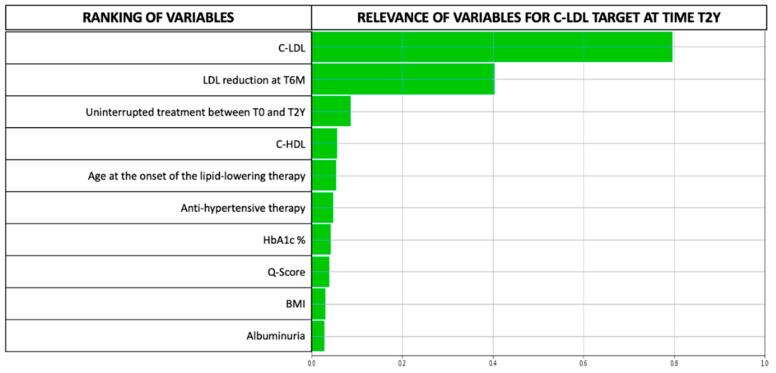
LLM-model with the FR of the most relevant variables predicting achievement of the C-LDL goal at T2Y.

Out of the 92 variables considered in the LLM, ten were found to be particularly relevant for discriminating the likelihood of lipid goal attainment within two years and were further analysed. The remaining variables in the database were deemed either not significant or only marginally so (Figure 5). The LLM model’s performance was evaluated based on precision, accuracy, and recall, with values of 0.78, 0.69, and 0.70 respectively. We also calculated the F1 Score, which takes into account both false positives and false negatives and provides a reliable measure of the model’s performance, especially for datasets with imbalanced classes, such as ours. The F1 Score, which ranges from 0 (poor performance) to 1 (perfect classification), was 0.74 for our model. [32] Overall, these metrics indicate that the LLM model performed reasonably well in classifying variables. To further validate the power of the LLM model in predicting C-LDL target achievement at time T2Y, we constructed the receiver operating characteristic (ROC) curve and calculated the area under the curve (AUC), which was equal to 0.79, *p* < 0.001 (see Figure S3, Supplementary Materials). Figure 6 presents the feature ranking (FR) and the relevance of the features automatically identified by the LLM for the model generated. Interestingly, the LLM determined that the LDL-C value at the beginning of lipid-lowering therapy and its value at the six-month follow-up visit had the most significant influence on achieving the primary objective. While these variables were deemed most critical, the LLM identified other predictors of LDL-C target achievement, albeit with a marginal role. These variables included uninterrupted treatment between T0 and T2Y, baseline BMI, micro-albuminuria and HDL-C levels, younger age at the start of lipid-lowering therapy, anti-hypertensive drug administration, lower glycosylated haemoglobin levels, and a higher Q-score. It is noteworthy that the response to hypolipidemic therapy does not vary significantly based on the type of antidiabetic treatment. Supplementary Table S2 shows the proportion of patients taking each specific antidiabetic medication in relation to our primary outcome. As previously described [33], the length of the bars in Figure 5 indicates the relative importance of each variable.

**Figure 7:**
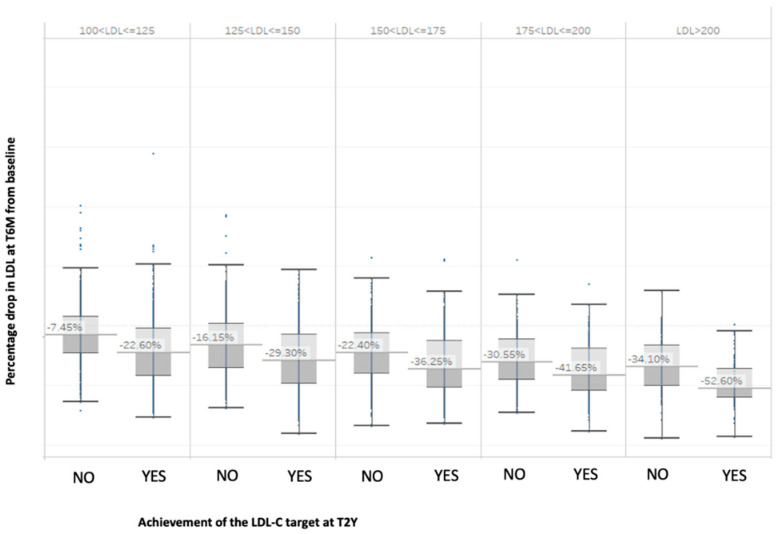
Percentage reduction in LDL-C at T6M compared to baseline based on LDL-C ranges and divided by target attainment at T2Y.

To verify these results, we performed a subgroup analysis applying the LLM algorithm to each group of patients with different ranges of LDL-C at the initiation of cholesterol-lowering therapy. A predictive model was consequently created for each of these subgroups (See Figure S4 in the Supplementary Materials). These new models, all with AUC-ROC between 0.78 and 0.88, confirmed that the 6-month LDL-C reduction was the most significant variable in predicting therapeutic success. The percentage reduction in LDL-C levels after 6 months (T6M) compared to baseline varies among different patient groups based on their LDL-C ranges and whether they achieved their target LDL-C levels after 2 years (T2Y). Specifically, we observed that patients with higher baseline LDL-C levels had a greater percentage reduction in LDL-C levels at T6M compared to those with lower baseline LDL-C levels. Furthermore, patients who achieved their target LDL-C levels at T2Y had a higher percentage reduction in LDL-C levels at T6M compared to those who did not reach their targets. These results are presented in Figure 7.

Furthermore, the AI models identified specific ‘threshold values’ for reducing LDL-C levels that, if attained within six months of initiating therapy, can predict the probability of achieving the treatment objective by the 2-year follow-up. These threshold values are listed in Table 3.

**Figure 8:**
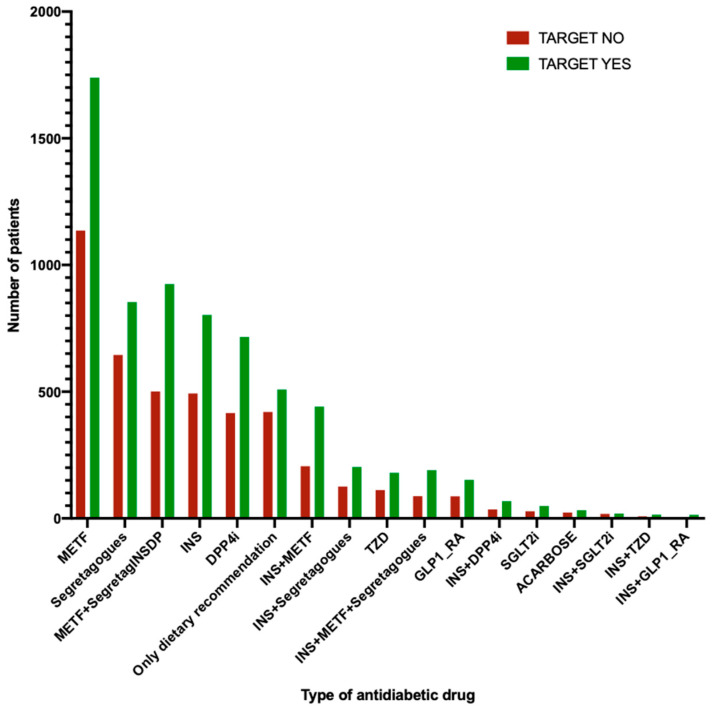
Distribution of patients achieving and not achieving the LDL-C target by type of antidiabetic prescribed.

Finally, we conducted a subgroup analysis to assess whether antidiabetic therapy, although not identified as a significant parameter by our machine learning approach, could still exert influence on the outcome. Supplementary Table S2 presents the distribution of patients, along with their glycated haemoglobin and LDL-C levels at baseline, categorized by type of antidiabetic therapy. By performing the chi-square test on the contingency table encompassing all the different types of antidiabetic agents, we found a statistically significant difference in the distribution of patients who met the target at T2Y compared to those who did not, regardless of the specific type of antidiabetic therapy received (Χ2 = 67.73, *p* < 0.0001), as illustrated in Figure 8. Furthermore, we observed that patients who did not achieve the LDL-C target at T2Y had significantly higher LDL-C levels at baseline in comparison to those who reached the target (please refer to Supplementary Figure S5b). This finding reinforces the significance of LDL levels at baseline (T0) in predicting the response to dyslipidemia therapy, as highlighted by our machine learning analysis.

Regarding glycated haemoglobin, we identified a statistically significant difference at baseline solely within the group of patients treated with glucagon-like peptide-1 agonists (GLP1-ra), as indicated in Supplementary Figure S5a.

## 4. Discussion

To the best of our knowledge, this is the first study providing real-world data from a very large population of T2DM patients and determining the variables that contribute to achieving the LDL-C target two years after the start of therapy by leveraging a machine-learning approach.

The results from our cohort show that men are more likely to reach the expected LDL-C target than women. These findings agree with previous studies suggesting gender differences in response to lipid-lowering therapies [34,35].

This disparity in achieving lipid targets between men and women can be attributed to several factors. Following menopause, the decline in oestrogen levels in women can lead to unfavourable alterations in their lipid profiles, posing challenges in attaining target levels. [36] Furthermore, it is plausible that men may exhibit additional CV risk factors that warrant more aggressive treatment for dyslipidemia, compared to women. As a result, men may demonstrate a statistically significant higher rate of achieving optimal LDL-c levels [37].

Furthermore, it is already known that patients with higher cholesterol levels at baseline are more likely to experience a greater reduction in cholesterol levels with statin treatment, and our machine learning analysis confirmed this finding. However, our study outlined that the reduction in LDL-C six months after the start of drug treatment holds the second position among the 10 parameters identified by AI, thus representing a crucial factor in predicting the response to treatment. In this regard, as presented in Figure S2 of the Supplementary Materials, we found that patients who do not achieve the target treatment goal at T2Y may experience an unexpected increase in their LDL-C levels after T6M. These findings underline the importance of maintaining hypolipidemic therapy over time and the need for implementing interventions that enhance patient adherence to treatment protocols for achieving and sustaining optimal LDL-C levels.

Our results are innovative as artificial intelligence was able to identify, for each baseline C-LDL, the minimum reduction that needs to be achieved by the next six-month visit to increase the likelihood of reaching the therapeutic goal within two years. Interestingly, these percentages differ from the average C-LDL cholesterol reduction calculated using classical statistical methods (as shown in Figure 7). These values can serve as a practical and easy-to-use tool for physicians to customize therapy and make appropriate therapeutic changes based on these predictions. In addition, as the guidelines do not specify a particular time frame for the reassessment of C-LDL levels in dyslipidemic patients following the initiation of therapy, our findings suggest that physicians should consider scheduling follow-up assessments within six months of initiating therapy for all diabetic patients. This will allow for the monitoring of LDL-C reduction and statin adherence.

The third predictor of treatment success identified by machine learning is an uninterrupted treatment between T0 and T2Y, confirming that some patients do not achieve adequate LDL-C reduction due to non-adherence. Therefore, asking about patients’ compliance with treatment during follow-up visits is essential.

Our predictive model also indicated that patients with lower levels of glycated haemoglobin and receiving pharmacological treatment for hypertension have a higher likelihood of reaching the C-LDL target two years after starting therapy. This result was unsurprising, as dyslipidemia is caused by the interplay of inflammatory processes and the accumulation of energy-rich substrates such as glucose and free fatty acids in the body. This metabolic imbalance leads to excessive production of lipoproteins in the liver and intestines, exacerbating dyslipidemia [38]. Moreover, it is worth mentioning that some classes of antihypertensives, such as those acting on the renin–angiotensin–aldosterone system, the alpha1 antagonists, and the calcium channel blockers, may have a favourable metabolic impact in hyperlipidemic patients. The alpha1-blockers, for example, have shown a reduction in total cholesterolemia and triglycerides and an increase in HDL-cholesterol values [39]. These effects are mediated by the rise in the number of LDL receptors, a reduction in HMG Co-A reductase activity, a decrease in VLDL synthesis, and increased lipoprotein lipase activity [40]. So far, several trials have reported that concomitant treatment of hypertension and dyslipidemia results in a significantly greater decrease in the risk of developing ASCVD [41,42].

In addition, our LLM analysis revealed that BMI, HDL-C, and albuminuria at T0 are also predictive variables for achieving the LDL-C goal at T2Y. However, the AI algorithm was unable to provide specific thresholds for these variables. As suggested by other authors, a possible explanation for the relationship between HDL levels and the success of hypolipidemic treatment could be that statins reduce C-LDL values and, at the same time, increase apolipoprotein A-I synthesis and HDL neogenesis in the liver [43]. In addition, HDL has pleiotropic effects, including anti-inflammatory and antioxidative effects [44].

Regarding BMI, it is worth noting that diabetic individuals who are obese may exhibit a suboptimal response to statin therapy. This could be due to differences in lipid metabolism or liver function, as well as chronic inflammation that negatively impacts the effectiveness of these drugs [45]. Furthermore, a recent meta-analysis reported that the impact of statins is significantly greater in individuals with pathologic albuminuria, highlighting the potential benefits of statin therapy in this particular patient population [46].

Another factor that may play a role in the likelihood of achieving C-LDL therapeutic goals is the age at the start of hypolipidemic therapy. The model suggests that younger patients are more likely to reach the treatment goal at T2Y. Our finding is not in line with what was reported in the PROSPER study, in which the non-responders to statin therapy are more likely to be younger patients who often have low disease awareness and are less adherent to medications [14]. This disparity may be attributed to the larger proportion of elderly individuals in our study cohort, who are more likely to present with a more severe dyslipidemic profile.

Furthermore, the quality of care provided to each patient, as indicated by the Q-score, has a positive impact on the success of therapy.

This study has several limitations that need to be considered. First, we were not able to include specific information on the dosage of the lipid-lowering medication regimen taken by patients, which is a factor that can certainly influence the achievement of therapeutic goals. However, the LLM analysis included the type of statin among the variables, and it was found that the trend in LDL-C evolution was similar across different statins, though they are known to vary in efficacy with the same dosage. Additionally, the type of drug was not identified as one of the most significant variables predicting the achievement of the primary outcome in the LLM analysis.

Secondly, it is important to note that we lacked information regarding the different classes of antihypertensive therapy used, though the presence of antihypertensive treatment was identified as a significant variable by our machine learning approach. Additionally, our dataset from the AMD database did not include genetic data, but since certain genetic variations have been linked to an increased or decreased response to statins, genotyping can provide valuable information about a patient’s likelihood of responding to these drugs [47].

Furthermore, while we implemented rigorous inclusion criteria for our study, it is important to acknowledge the potential limitation of selection bias. This bias may arise due to the inclusion of patients from specialized diabetes centres, which could result in the enrolment of individuals with a higher burden of CV risk factors. Additionally, it is worth noting that non-adherence to follow-up could be associated with non-adherence to therapy, introducing another potential bias to our analysis.

Moreover, our study focuses on an LDL-C target of less than 100 mg/dL (2.60 mmol/L) two years after initiation of hypolipidemic therapy, based on a cohort of patients who began treatment between 2002 and mid-2017, while the current guidelines suggest a more stringent treatment goal of an LDL cholesterol level below 70 mg/dL (1.8 mmol/L) for individuals with diabetes and at least one other cardiovascular disease risk factor, as well as a minimum reduction from their baseline LDL cholesterol level of 50 per cent [24]. In addition, some of the patients recruited until 2017 were being treated with antidiabetics that have now been replaced by new classes of drugs. Considering all these aspects, future prospective investigations are warranted to confirm our preliminary results and test whether the same predictive parameters are also identified for stricter LDL-C targets.

Lastly, the heterogeneity of the commercial assays used to assess cholesterol levels might have impacted the accuracy of the measurements, as biochemical parameters were not assessed in the same laboratory. Despite these limitations, we utilized AI to analyse real-world data in a significant patient cohort and identified novel parameters that can facilitate the differentiation between lipid-lowering therapy responders and non-responders, ultimately enabling more personalized treatment strategies.

## 5. Conclusions

The management of dyslipidemia in individuals with T2DM is still a significant challenge. The AI algorithm identified parameters that can account for the variation in response to lipid-lowering interventions. In addition, AI can assist in monitoring patient responses to treatment over time, enabling doctors to adjust treatment plans as necessary.

## Figures and Tables

**Table 1:** Demographic and metabolic characteristics of the study cohort at T0, T6M, and T2Y, broken down by C-LDL target achievement at T2Y.

		T0			T6M		T2Y	
Variable	Achieved Target at T2Y	Mean	SD	*p*-Value	Mean	SD	Mean	SD
Age	NO	65.06	10.03	<0.001	65.55	10.03	67.05	10.02
(y)	YES	65.96	9.49		66.47	9.5	67.96	9.49
HbA1c	NO	7.29	1.22	0.92	7.19	1.13	7.28	1.16
(%)	YES	7.28	1.21		7.2	1.11	7.21	1.06
LDL-C	NO	142.64	32.26	<0.001	117.08	32.5	127.52	23.72
(mg/dL)	YES	120.86	36		88.87	30.65	74.35	16.01
HDL-C	NO	51.75	13.08	<0.001	51.01	13.33	51.18	12.98
(mg/dL)	YES	50.15	13.39		49.54	13.6	49.47	14.04
TGD	NO	143.6	73.43	0.32	135.74	77.61	139.33	69.97
(mg/dL)	YES	142.62	72.49		129.05	64.41	125.08	66.23
BMI	NO	29.64	5.29	0.11	29.6	5.23	29.6	5.3
(Kg/m^2^)	YES	29.79	5.24		29.77	5.25	29.71	5.3
eGFR	NO	79.09	20.3	<0.001	78.36	20.36	76.66	20.9
(mL/min/m^2^)	YES	76.92	20.47		76.34	20.69	75.34	21.67
Glucose	NO	147.01	44.4	0.12	143.39	43.69	144.89	43.96
(mg/dL)	YES	148.07	45.28		143.05	40.05	142.05	42.95
DBP	NO	79.75	9.58	0.08	79.26	9.62	78.83	9.62
(mmHg)	YES	79.39	9.44		78.23	9.15	77.76	9.22
SBP	NO	137.89	18.21	0.39	137.28	17.99	137.72	18.32
(mmHg)	YES	138.11	18.33		136.47	17.83	136.38	17.59
TC	NO	223.02	36.92	<0.001	195.08	37.54	206.57	29.81
(mg/dL)	YES	199.79	41.35		164.16	36.57	148.75	22.72
Uric A.	NO	5.51	1.96	0.05	5.47	1.46	5.53	1.55
(mg/dL)	YES	5.59	1.84		5.56	1.59	5.47	1.53

Abbreviations: SD, standard deviation; BMI, body mass index; HbA1c, glycated haemoglobin; eGFR, estimated glomerular filtration rate; SBP, systolic blood pressure; DBP, diastolic blood pressure; LDL-C, low-density lipoprotein cholesterol; TC, total cholesterol; TGD, triglycerides; HDL-C, high-density lipoprotein cholesterol; Uric A, uric acid; T6M, 6-month follow-up visit; T2Y, 2-year follow-up visit.

**Table 2:** Percentages of patients for each LDL-C interval at T0, T6M, and T2Y.

		LDL-C ≤ 75	75 < LDL-C ≤ 100	100 < LDL-C ≤ 125	125 < LDL-C ≤ 150	150 < LDL-C ≤ 175	175 < LDL-C ≤ 200	LDL > 200
at T0	Patients n (%)	959 (8.52%)	1509 (13.41%)	2307 (20.5%)	3266 (29.03%)	2207 (19.61%)	759 (6.75%)	245 (2.18%)
at T6M	Patients n (%)	2908 (25.84%)	3390 (30.13%)	2480 (22.04%)	1504 (13.37%)	686 (6.1%)	221 (1.96%)	63 (0.56%)
at T2Y	Patients n (%)	3363 (29.89%)	3607 (32.06%)	2370 (21.06%)	1222 (10.86%)	497 (4.42%)	141 (1.25%)	52 (0.46%)

Abbreviations: n, number; LDL-C, low-density lipoprotein cholesterol; T6M, 6-month follow-up visit; T2Y, 2-year follow-up visit.

**Table 3:** Threshold values at T6M suggested by machine learning.

Baseline LDL-C Range	Probable LDL-C Target After 2 Years: YES	Probable LDL-C Target After 2 Years: NO
100 < LDL-C ≤ 125	LDL-C reduction 6 months after T0 > 14%	LDL-C reduction 6 months after T0 < 14%
125 < LDL-C ≤ 150	LDL-C reduction 6 months after T0 > 32%	LDL-C reduction 6 months after T0 < 30%
150 < LDL-C ≤ 175	LDL-C reduction 6 months after T0 > 33%	LDL-C reduction 6 months after T0 < 33%
175 < LDL-C ≤ 200	LDL-C reduction 6 months after T0 > 45%	LDL-C reduction 6 months after T0 < 45%
200 < LDL-C ≤ 250	LDL-C reduction 6 months after T0 > 47%	LDL-C reduction 6 months after T0 < 47%

Abbreviations: LDL-C, low-density lipoprotein cholesterol.

## Data Availability

Data will be made available upon reasonable request to the corresponding author.

## Supplementary Materials

The following supporting information can be downloaded at: https://www.mdpi.com/article/10.3390/jcm12124095/s1, Figure S1: Type of lipid-lowering drugs prescribed during the inclusion period; Figure S2: The trend of LDL-C levels in patients who achieve/do not achieve the target at T2Y divided by the starting LDL-C range; Figure S3: ROC curve of the first LLM-model; Figure S4: Single LLM models with the most relevant variables predicting achievement of the C-LDL target at T2Y for each initial LDL-C range; Figure S5: Differences in baseline HbA1c and LDL-C among those patients who meet the LDL-C target at T2Y and those who do not, for each type of antidiabetic treatment; Table S1: Summary of variables included in the study; Table S2: Categorization of patients based on their anti-diabetic medication, divided into those who meet the target and those who do not.
