# Supplementary material for: Uncovering Predictors of Lipid Goal Attainment in Type 2 Diabetes Outpatients Using Logic Learning Machine: Insights from the AMD Annals and AMD Artificial Intelligence Study Group
Source: J Clin Med. 2023 Jun 16;12(12):4095. doi: 10.3390/jcm12124095 (PMC10299248; doi:10.3390/jcm12124095)
Supplement: Supplementary file 1 [file jcm-12-04095-s001.zip › jcm-2334064-supplementary.pdf]

Figure S1: Type of lipid-lowering drugs prescribed during the inclusion period

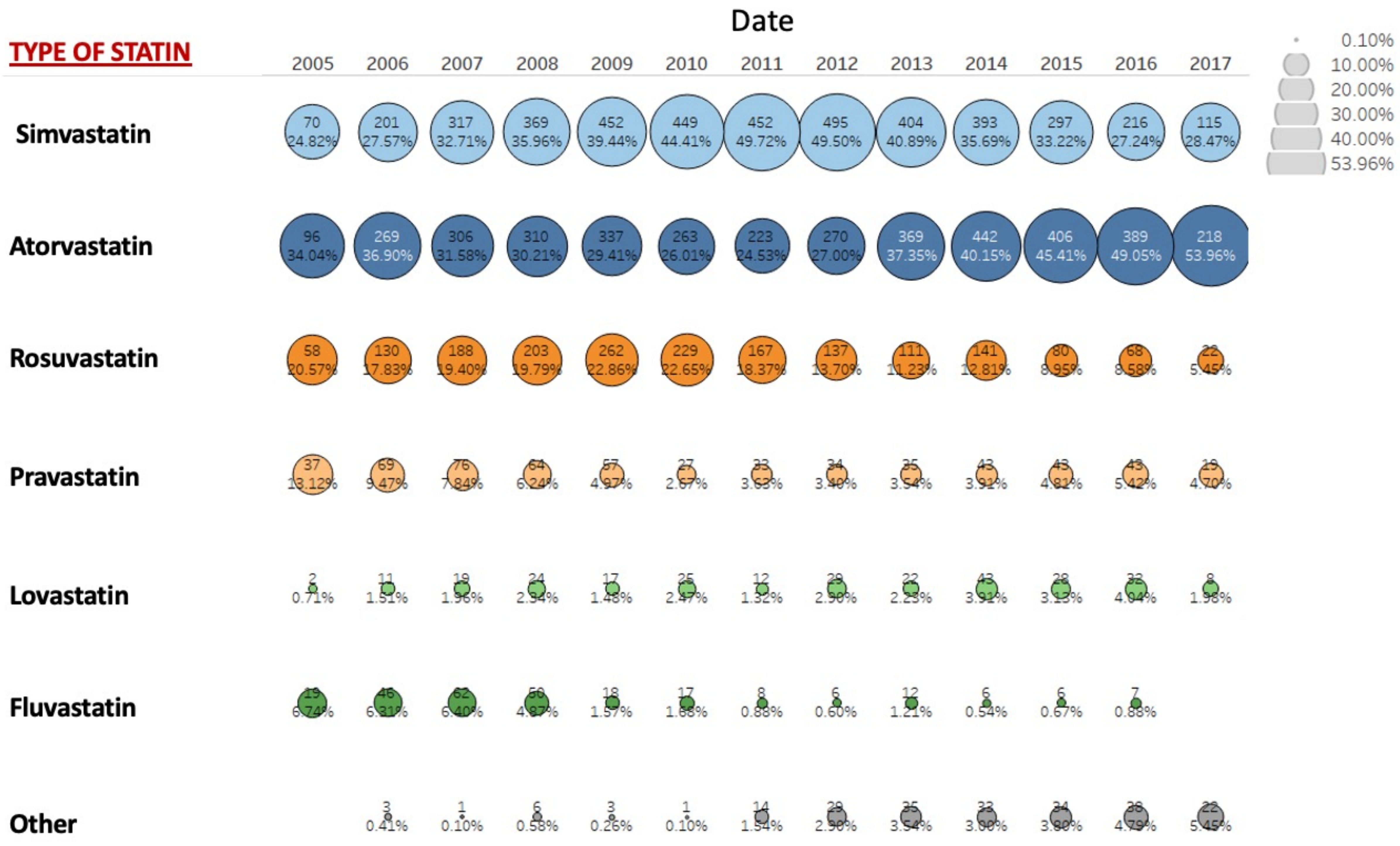

Figure S2: Analysis of LDL-C Level Trends in Patients who Achieve/Do Not Achieve Target LDL-C at Two-Year Follow-Up, Stratified by Initial LDL-C Range

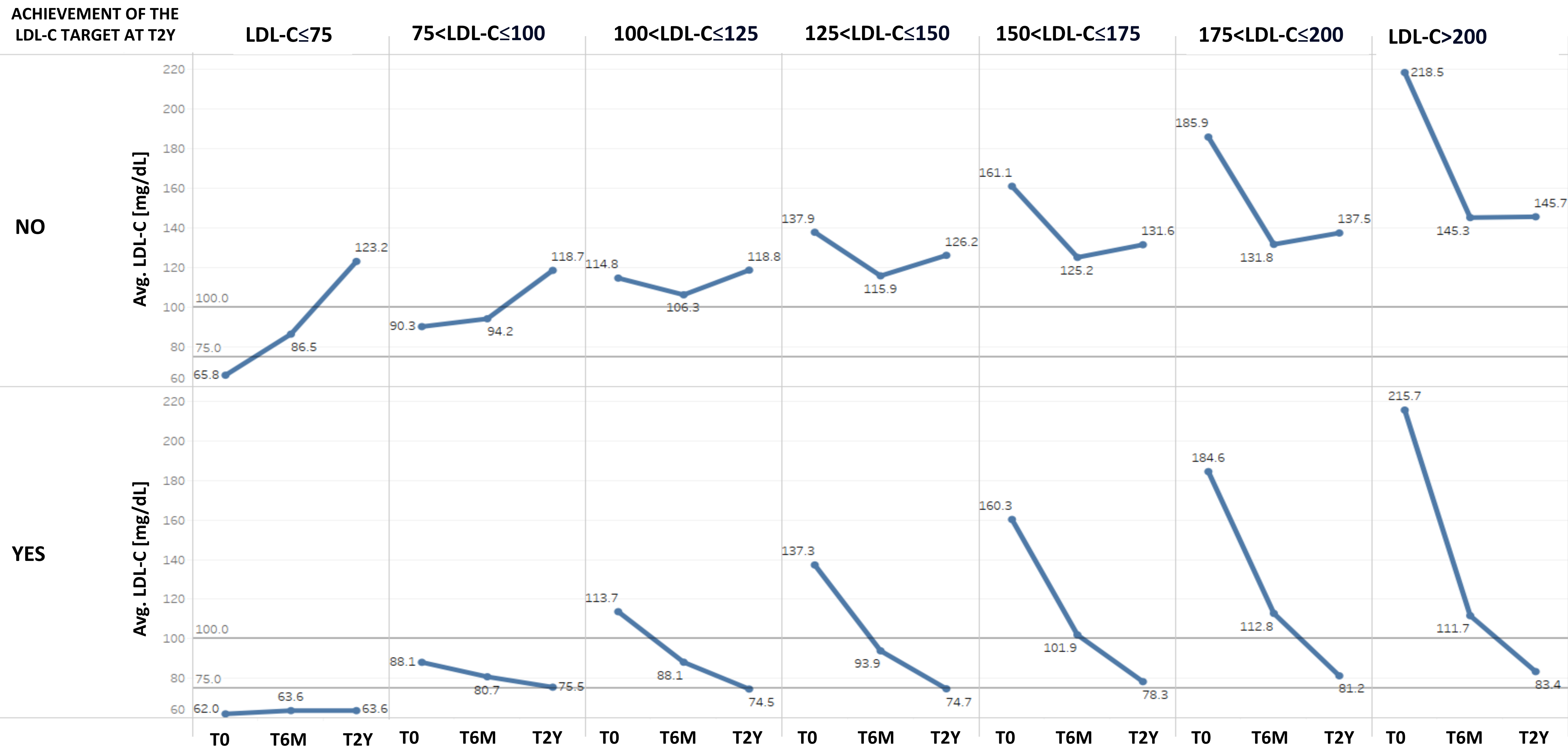

Figure S3: ROC-AUC for Logic Learning Machine Model Identifying the Most Relevant Predictive Variables for Achieving C-LDL Goal at T2Y (11.252 patients)

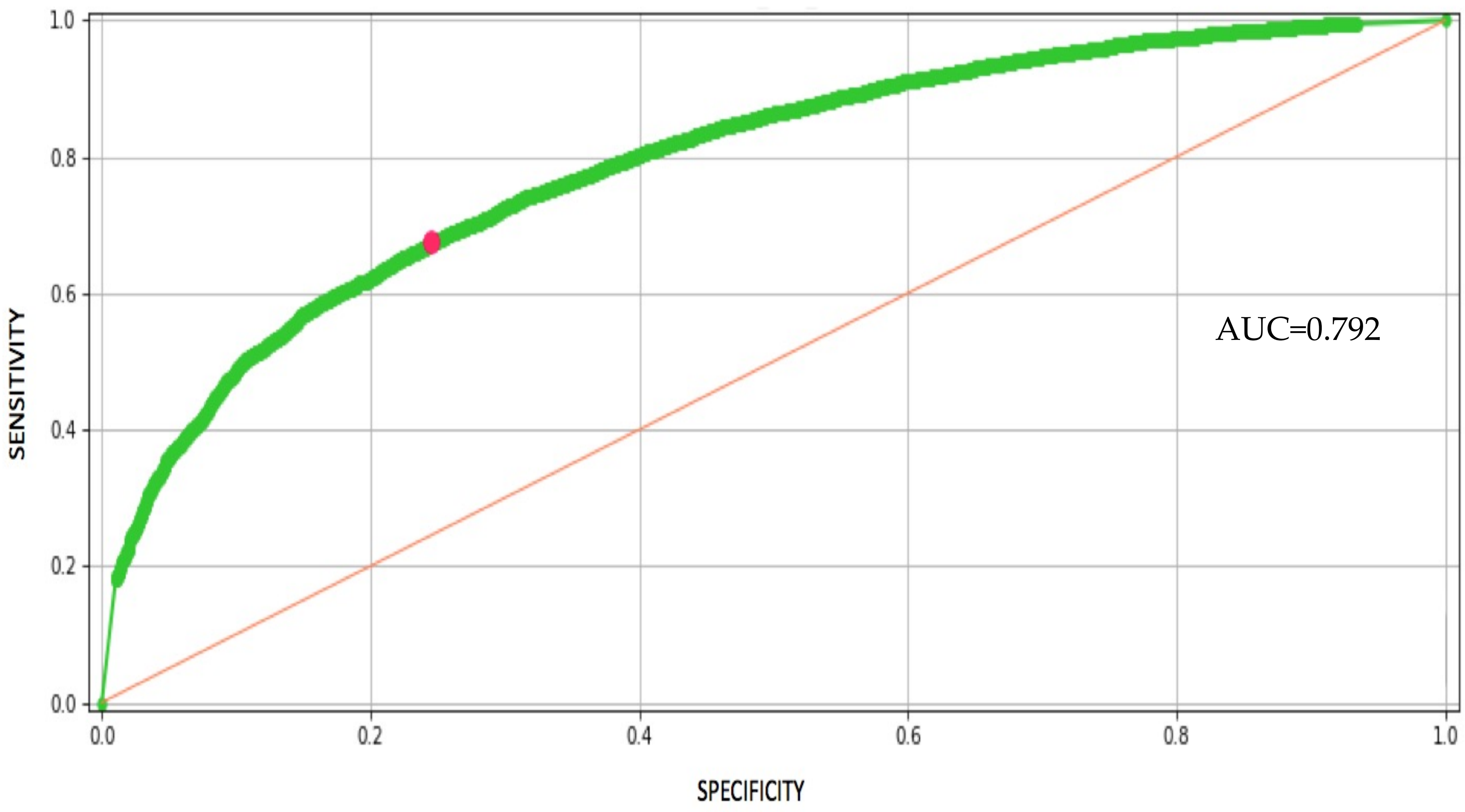

Figure S4: Specific LLM Models with the most relevant variables predicting achievement of the LDL-C target at T2Y for each initial LDL-C range

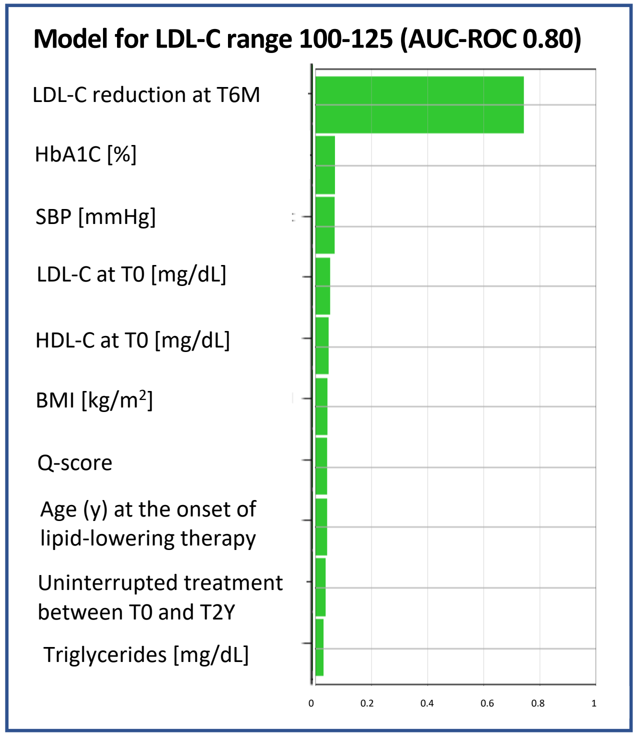

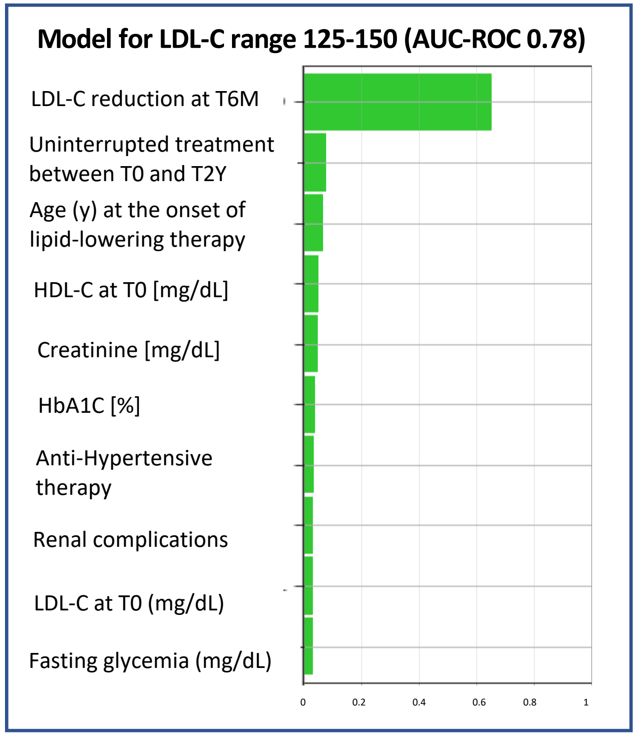

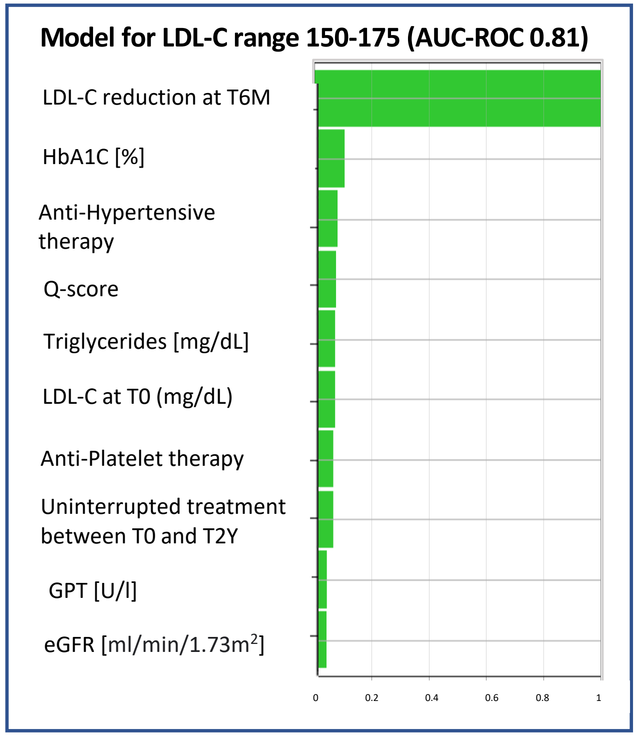

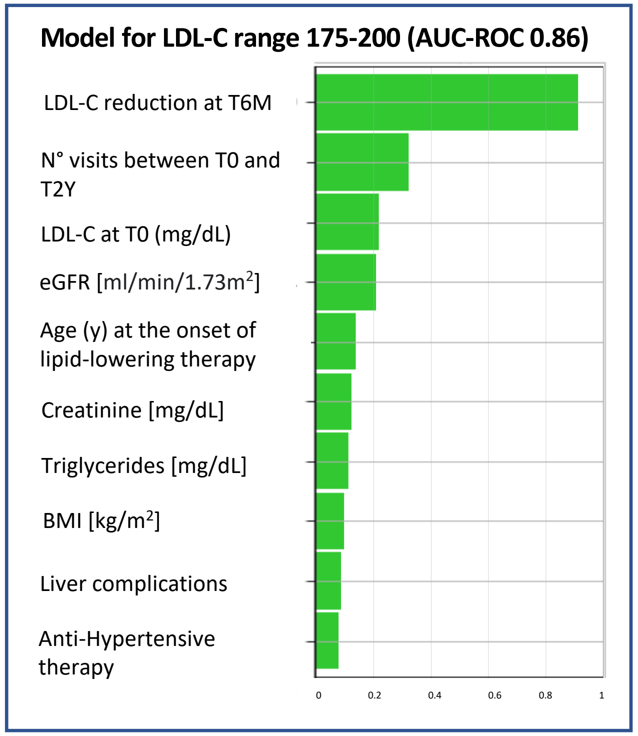

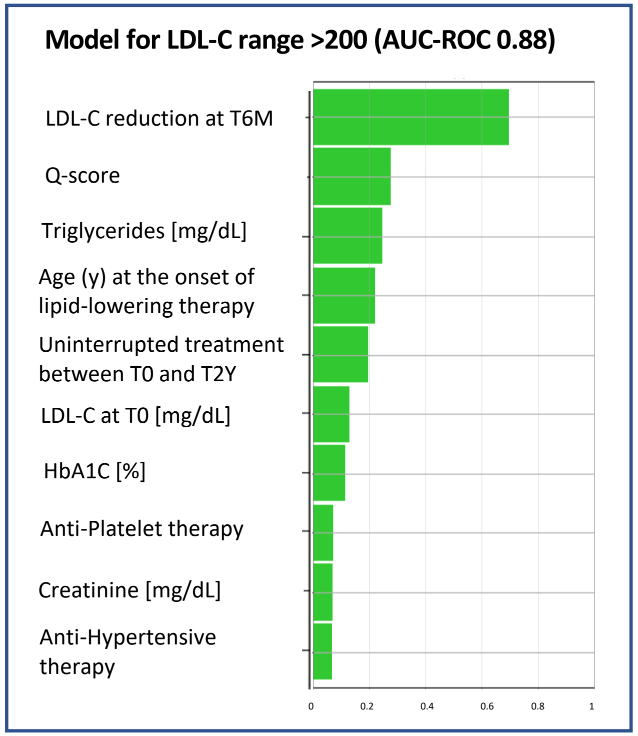

Table S1: Summary of variables included in the study

•ANTROPOMETRIC PARAMETERS	•ORGANIZATIONAL PARAMETERS
Age;	Q-score;
Sex;	Adherence to treatment (self-reported);
Weight;	Years of clinical observation,
Height;	(considered a proxy of duration of diabetes);
Waist circumference;	**•TREATMENTS**
Hip circumference;	Drug therapy for diabetes (type and associations);
Body mass index (BMI);	Drug therapy for dyslipidemia (type and associations);
Systolic blood pressure (BP), diastolic BP;	Drug therapy for hypertension (present or not);
and derived variables or index.	Additional drugs.
**•BIOCHEMICAL PARAMETERS**	**•DIABETES COMPLICATIONS AND COMORBIDITIES**
HbA1c at current visit;	Presence of nephropathy;
fasting glucose;	Presence of atrial fibrillation;
Triglycerides;	History of heart failure;
Total cholesterol;	History of stroke;
High-density lipoproteins (HDL);	History of cardiac complications;
low-density lipoproteins (LDL);	Presence of vasculopathy;
Creatinine;	Presence of lower limb complications;
estimated glomerular filtration rate (eGFR;	Presence of neuropathy;
micro–macro/albuminuria;	Presence of foot complications;
serum uric acid;	Presence of eye complications;
serum glutamic oxaloacetic transaminase (GOT);	Presence of hepatopathy.
serum glutamic pyruvic transaminase (GPT);	
and same variables for follow-up visits.	

Table S2: Categorization of patients based on their anti-diabetic medication, divided into those who meet the target and those who do not

	TARGET LDL-C at T2Y							
	NO				YES			
TREATMENT	Number (n)	Mean HbA1C (%) at T0	Mean LDL-C at T0	% of patients	Number (n)	Mean HbA1C (%) at T0	Mean LDL-C at T0	% of patients
METF	1136	6,86	145,23	26,2	1739	6,81	125,09	25,2
Segretagogues	645	7,24	145,70	14,9	854	7,24	124,70	12,4
METF+Segretagogues	501	7,53	138,24	11,5	925	7,43	117,76	13,4
INS	493	7,90	141,78	11,4	803	7,86	115,48	11,6
DPP4i	416	7,29	138,25	9,6	716	7,28	115,02	10,4
Only dietary recommendation	420	6,36	147,20	9,7	509	6,37	128,12	7,4
INS+METF	206	8,28	139,57	4,7	441	8,07	115,68	6,4
INS+Segretagogues	126	8,09	136,84	2,9	203	8,03	118,58	2,9
TZD	112	7,45	142,70	2,6	180	7,26	132,94	2,6
INS+METF+Segretagogues	88	8,21	137,58	2	190	8,07	118,33	2,8
GLP1_RA	87	7,65	135,35	2	152	7,36	116,89	2,2
INS+DPP4i	35	7,73	126,86	0,8	68	7,72	110,87	1
SGLT2i	28	7,40	148,81	0,6	49	7,17	121,16	0,7
ACARBOSE	23	6,82	164,61	0,5	32	6,79	123,78	0,5
INS+SGLT2i	18	9,02	144,33	0,4	19	8,05	98,82	0,3
INS+TZD	8	7,90	134,65	0,2	15	8,57	117,72	0,2
INS+GLP1_RA	1	6,50	102,00	0	14	8,11	102,41	0,2
**Total**	4343				6909			

Abbreviations: n, number; METF, metformin; INS, insulin; TZD, thiazolidinediones, GLP1_RA, Glucagon-like peptide-1 agonists; SGLT2i, The sodium glucose cotransporter 2 inhibitors; DPP4i, Dipeptidyl peptidase 4 inhibitors

Figure S5: Differences in baseline HbA1c and LDL-C among those patients who meet the target of LDL-C at T2Y and those who do not, for each type of antidiabetic treatment

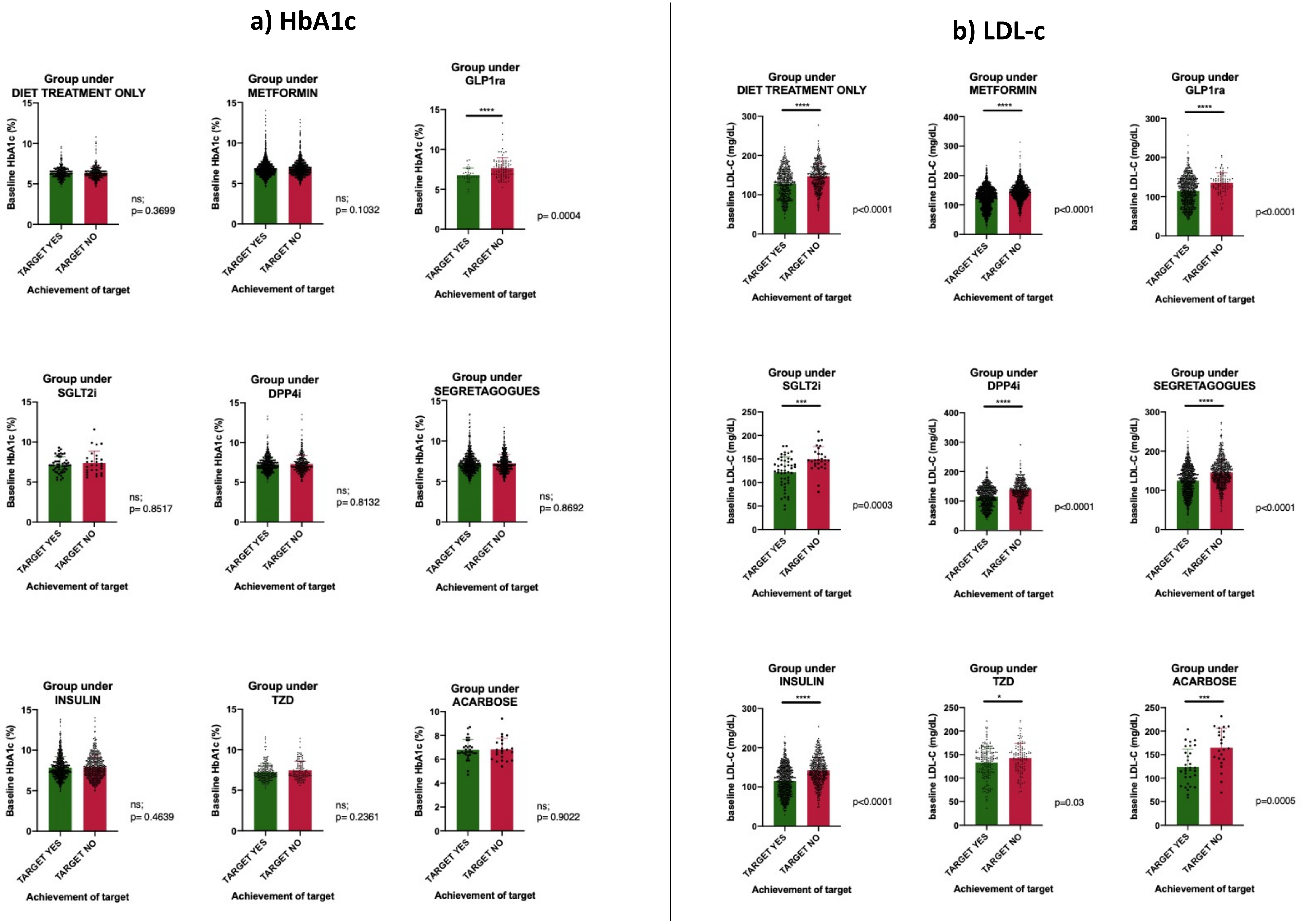

Abbreviations:; INS, insulin; TZD, thiazolidinediones, GLP1ra, Glucagon-like peptide-1 agonists; SGLT2i, The sodium glucose cotransporter 2 inhibitors; DPP4i, Dipeptidyl peptidase 4 inhibitors
